# RAB20 deficiency promotes the development of silicosis *via* NLRP3 inflammasome

**DOI:** 10.3389/fimmu.2022.967299

**Published:** 2022-09-05

**Authors:** Zhouyangfan Peng, Mingwu Duan, Kai Zhao, Yiting Tang, Fang Liang

**Affiliations:** ^1^ Department of Hematology and Critical Care Medicine, The 3rd Xiangya Hospital, Central South University, Changsha, China; ^2^ Department of Physiology, School of Basic Medical Science, Central South University, Changsha, China

**Keywords:** NLRP3, RAB20, pulmonary fibrosis, silicosis, lysosomal damage

## Abstract

Silicosis is a worldwide serious occupational disease that is caused by inhalation of silica crystals. However, little is known about the pathogenesis mechanism of silicosis. We performed single-cell sequencing in bronchoalveolar lavage fluid (BALF) from mine workers with silicosis and their co-workers who did not develop silicosis, and found that the RAB20 deficiency in monocytes/macrophages was strongly linked to the development of silicosis. In the silicosis murine model, RAB20 knockout markedly enhanced the silica crystal-induced pulmonary interstitial fibrosis and respiratory dysfunction. Moreover, this process is strongly accompanied by IL-1β release and NLRP3 activation. *In vitro*, RAB20 knockout macrophages aggravated the crystalline silica-induced IL-1β release and NLRP3 inflammasome activation partly by increased ratio of crystalline silica/phagosomal areas/volumes to induce lysosomal injury. Thus, these findings provide novel molecular insights into the intricate mechanisms underlying lysosomal protein RAB20 that are necessary for environmental irritant-mediated innate immunity, and shed light on the future development of novel therapy target for the prevention of silicosis.

## Introduction

Silicosis is a chronic interstitial pulmonary disease that is induced by the inhalation of free silica crystal dust (1), and is characterized by granulomatous lung inflammation, delayed pulmonary interstitial fibrosis, and progressive respiratory dysfunction (2, 3). Nowadays, silicosis remains one of the most populational occupational diseases in the world especially in China (3). China with over 500,000 silicosis patients has more than 6,000 new cases of silicosis each year and more than 24,000 deaths annually (4). As a major problem for workers in small-scale mines, silicosis is irreversible and not therapeutically curable. However, only a small proportion of workers with prolonged inhalation of free crystalline silica eventually develop silicosis (5, 6). It was previously believed that the susceptibility of silicosis might be due to individual genetic variation (7). Some studies report that the polymorphisms in genes encoding proinflammatory cytokines or growth factors are associated with the occurrence of silicosis (8, 9). However, the functional relevance of the gene polymorphisms to the development of silicosis has not been validated (10, 11). Thus, the underlying mechanisms of mammalian susceptibility to silicosis remain largely unknown.

Accumulating evidence shows that deregulated pulmonary immune responses drive the development and progression of silicosis (12, 13). Alveolar macrophages orchestrate lung inflammation in response to both infectious and non-infectious agents. Phagocytosis of the inhaled crystalline silica by alveolar macrophages results in the engulfment of inhaled particles into the phagosome and lysosomal damage, which elicits the activation of NOD-like receptor family pyrin domain-containing 3 (NLRP3) inflammasome, an intracellular protein complex that mediates the caspase-1 activation through apoptosis-associated speck-like protein containing CARD (ASC) and the subsequent proinflammatory cytokines including interleukin (IL)-1β and IL-18 maturation (14–16). In murine silicosis models, deletion of the genes encoding NLRP3 inflammasome components markedly attenuates silica crystal-induced granulomatous lung inflammation and pulmonary fibrosis (15, 16).

To investigate the lysosome injury-mediated activation mechanism of NLRP3 in silicosis, we analyzed single-cell RNA sequencing analysis of cells from bronchoalveolar lavage fluid (BALF) obtained from small-scale mine workers with or without silicosis. This led to the finding that the decreased level of RAB20 in monocytes/macrophages may be strongly associated with NLRP3 inflammasome activation and the occurrence of silicosis. RAB20 as a Rab GTPase regulates phago-lysosomal membrane trafficking by controlling cargo selection, vesicle budding, motility, tethering, and fusion. Although RAB20 has been associated with many components of the endocytic pathway, its precise role in regulating the silica crystal phagocytosis-mediated NLRP3 activation has not yet been investigated. In addition, using a murine silicosis model, we show that the RAB20 knockout greatly promoted a crystalline silica-induced silicosis-like picture through enhancing the NLRP3 inflammasome activity and IL-1β release. These results extend our understanding of how lysosomal protein regulates host responses to environmental irritants and suggest that decrease of RAB20 might be a high-risk factor for the development of silicosis.

## Results

### Reduced RAB20 in monocytes and macrophage was strongly associated with the occurrence of silicosis in mine workers

**Figure 1:**
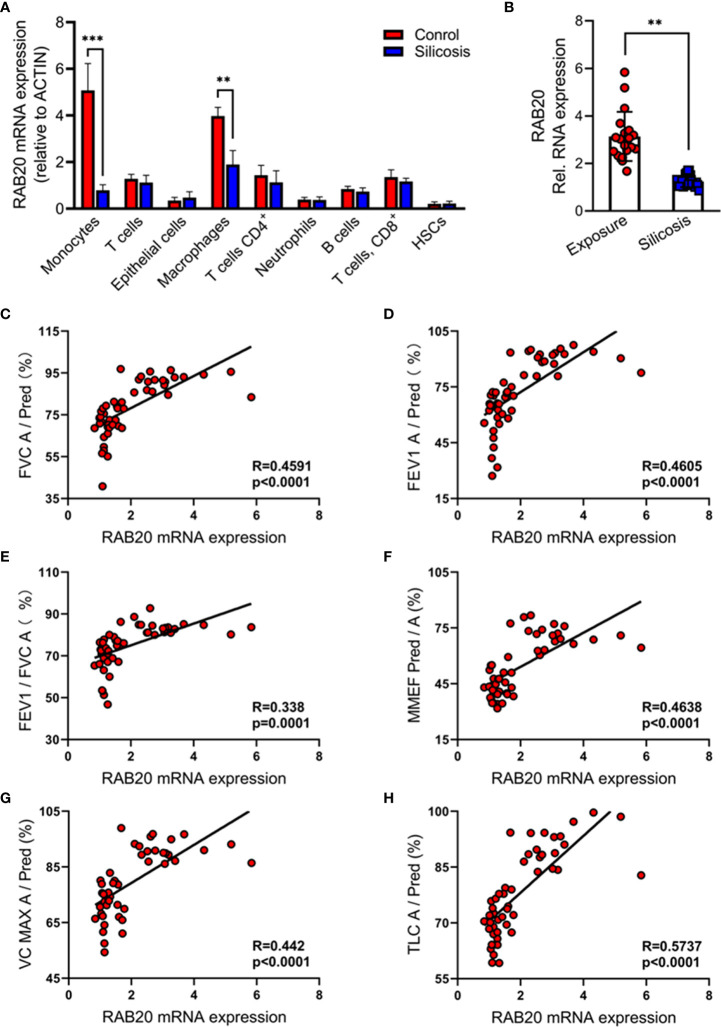
Reduced RAB20 in monocytes and macrophage was strongly associated with the occurrence of silicosis in mine workers. **(A)** Expression of RAB20 genes in monocytes, T cells, macrophages, CD4^+^ T cells, neutrophils, CD8^+^ T cells, B cells, and HSCs based on publicly available single-cell RNA-seq data using bronchoalveolar lavage fluid cells obtained from silicosis patients and their healthy co-workers. **(B)** The RAB20 mRNA expression detected by qPCR in bronchoalveolar lavage fluid cells from exposure and silicosis patients. **(C)** The mRNA levels of RAB20 significantly correlated with the lung function indexes (actual FVC/predicted FVC) in silicosis patients. **(D)** The mRNA levels of RAB20 significantly correlated with the lung function indexes (actual FEV1/predicted FEV1) in silicosis patients. **(E)** The mRNA levels of RAB20 significantly correlated with the lung function indexes (actual FEV1/actual FVC) in silicosis patients. **(F)** The mRNA levels of RAB20 significantly correlated with the lung function indexes (actual MMEF/predicted MMEF) in silicosis patients. **(G)** The mRNA levels of RAB20 significantly correlated with the lung function indexes (actual VC MAX/predicted VC MAX) in silicosis patients. **(H)** The mRNA levels of RAB20 significantly correlated with the lung function indexes (actual TLC/predicted TLC) in silicosis patients. **p < 0.01; ***p < 0.001 (by unpaired/two-tailed t test or one-way and two-way ANOVA test). Data are shown as mean ± SEM from three independent experiments.

We analyzed publicly available single-cell RNA sequencing (scRNA-seq) data of bronchoalveolar lavage fluid cells, which are obtained from the silicosis patients and their healthy co-workers (17). We found that the expression of RAB20 genes is markedly decreased in monocytes and macrophages from silicosis patients, while the RAB20 gene expression in T cells (CD3^+^), CD4^+^ T cells (CD3^+^ and CD4^+^), CD8^+^ T cells (CD3^+^ and CD8^+^), monocytes (CD14^+^), macrophages (CD68^+^), B cells (B220^+^), and hemopoietic stem cells (HSCs, CD34^+^) from silicosis patients was not altered (**Figure 1A**). We also confirmed this observation that RAB20 was notably lower in monocyte/macrophage cells from silicosis patients using real-time quantitative PCR (**Figure 1B**). The development of silicosis correlates with progressive loss of lung function, which is characterized by a decrease in forced vital capacity (FVC), forced expiratory volume in 1 s (FEV1), the ratio of FEV1 to FVC, maximum midexpiratory flow (MMEF), vital capacity maximum (VC MAX), and total lung compliance (TLC). To determine if loss of RAB20 genes was associated with respiratory dysfunction, we analyzed the correlation between the expression of RAB20 gene expression and lung function. Decrease of RAB20 gene was significantly correlated with deteriorative FEV1, FVC, FEV1/FVC, MMEF, VC MAX, and TLC (**Figures 1C–H**). Together, these observations establish that RAB20 gene deficiency in lung monocytes/macrophages was strongly associated with the occurrence of silicosis.

### RAB20 deficiency promotes silica crystal-induced pulmonary interstitial fibrosis and respiratory dysfunction

**Figure 2:**
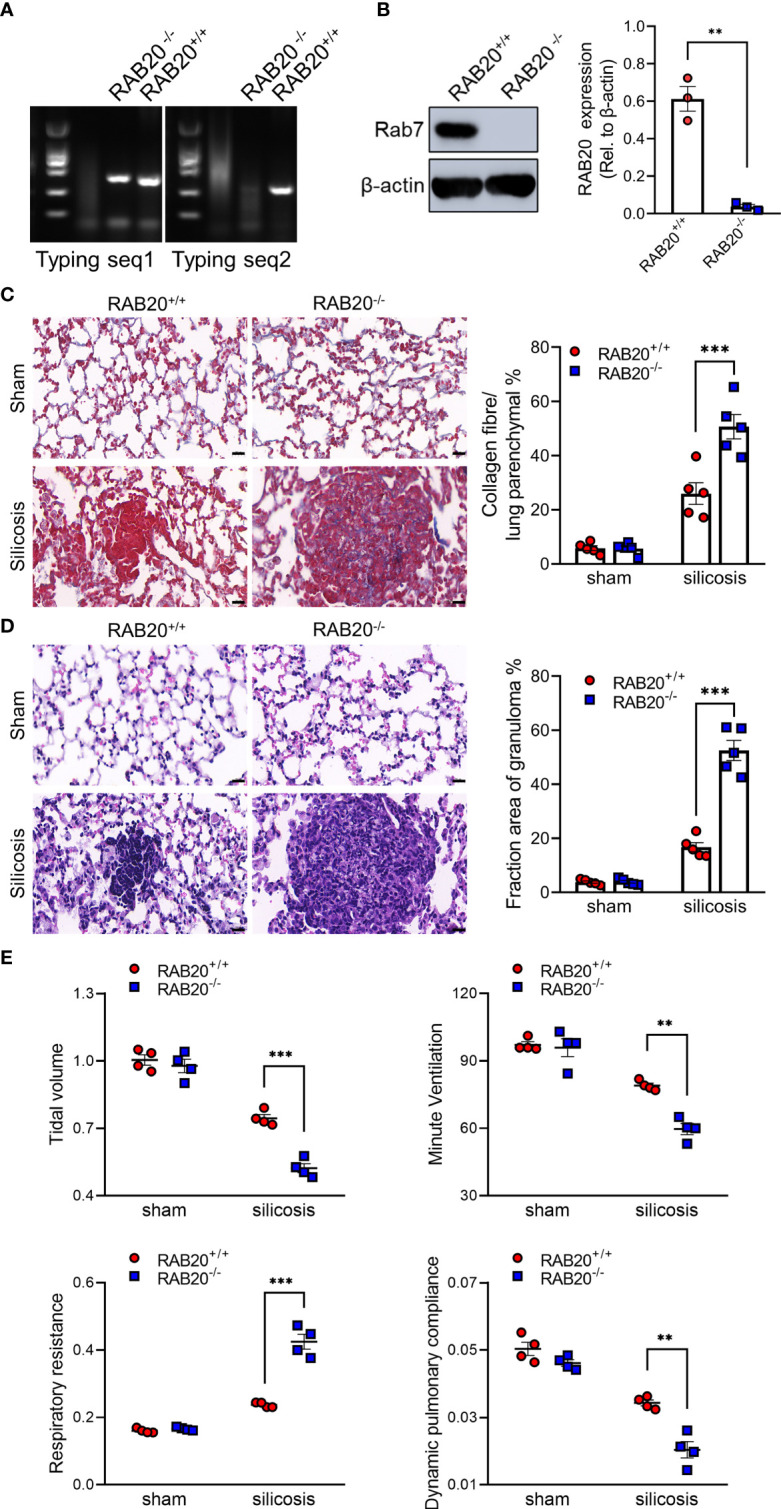
RAB20 deficiency promotes silica crystal-induced pulmonary interstitial fibrosis and respiratory dysfunction. **(A)** Typing lane of RAB20 in wild type and RAB20 knockout mouse. **(B)** Immunoblotting for RAB20 and β-actin in the lung tissues of wild type and RAB20 knockout mouse. **(C)** H&E staining of lung tissues in wild type or RAB20 knockout mice with or without silica crystals. Morphometry analysis quantifying granuloma area in the lungs. **(D)** Masson staining of lung tissues in wild type or RAB20 knockout mice inhaled with or without silica crystals. Morphometry analysis quantifying collagen fiber area in the lungs. **(E)** Wild type or RAB20 knockout mice were subjected to lung function assessment. Resistance index (RI), dynamic compliance (Cdyn), tidal volume (TV), and minute volume (MV) were measured. **p < 0.01; ***p < 0.001 (unpaired/two-tailed t test, one-way and two-way ANOVA test). Data are shown as mean ± SEM from three independent experiments. Scale bar represents 50 μm.

To test the functional relevance of RAB20 deficiency to the development of silicosis, we intratracheally injected a silica crystal suspension in wild-type (WT) mice and RAB20-deficient mice (**Figures 2A, B**) to establish a murine silicosis model. The degree of pulmonary interstitial fibrosis was visualized using hematoxylin–eosin (H&E) and Masson staining. As revealed by H&E and Masson staining, RAB20-deficient mice exhibited many more contiguous fibrotic masses and air bubbles, and greater fibrous obliteration as compared to RAB20 knockdown mice and their WT controls (**Figures 2C, D**), indicating that deletion of RAB20 markedly promoted silica crystal-induced pulmonary interstitial fibrosis. Moreover, the increased pulmonary interstitial fibrosis phenotype was associated with a decrease in dynamic compliance (Cdyn), tidal volume (TV), and minute volume (MV), and significantly increased respiratory resistance index (RI) in RAB20-deficient mice (**Figure 2E**). Together, these findings demonstrate that the RAB20 deficiency promotes silica crystal-induced pulmonary interstitial fibrosis and respiratory dysfunction, and establish a functional link between suppressed RAB20 expression and the susceptibility to silicosis.

### RAB20 deficiency promotes silica crystal-induced activation of NLRP3 inflammasome

**Figure 3:**
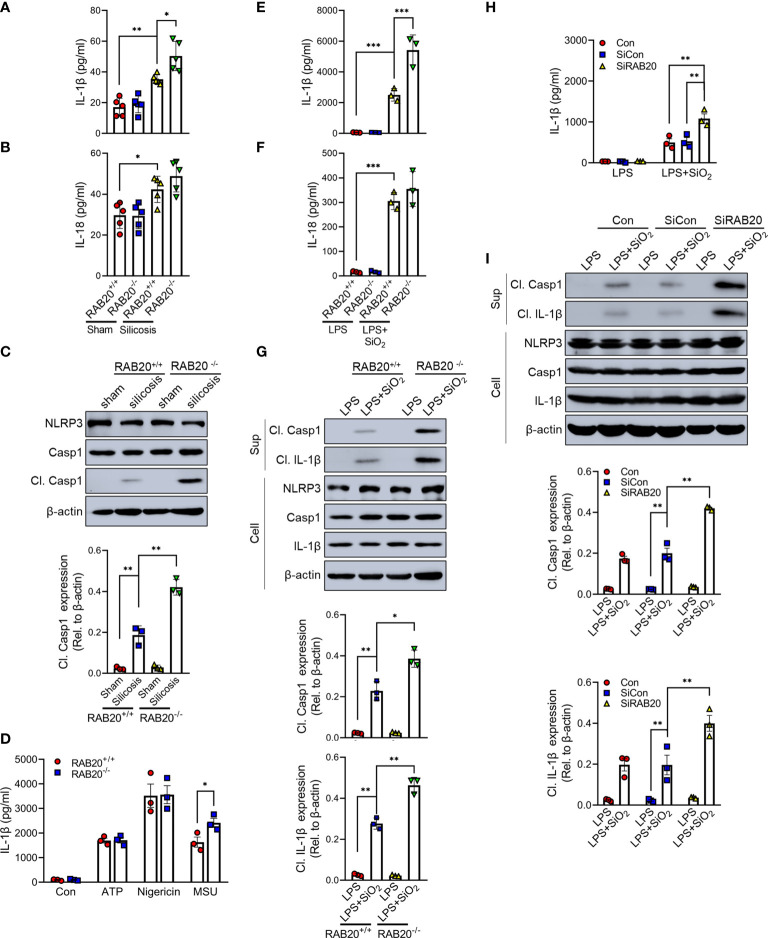
RAB20 deficiency promotes silica crystal-induced activation of NLRP3 inflammasome. **(A, B)** IL-1β and IL-18 levels in bronchoalveolar lavage fluid cells of wild type or RAB20 knockout mice with or without silica crystals. **(C)** Immunoblotting for caspase-1, NLRP3, and β-actin in the lung tissues of wild type and RAB20 knockout mouse stimulated with or without silica crystals. **(D)** IL-1β secretion in wild type and RAB20 knockout peritoneal macrophages stimulated with ATP, nigericin, and MSU after LPS priming. **(E, F)** IL-1β and IL-18 secretion in wild type and RAB20 knockout peritoneal macrophages stimulated with SiO_2_ after LPS priming. **(G)** Immunoblotting for IL-1β, caspase-1, NLRP3, and β-actin in the supernatants (SN) or cell lysates (cell) of wild type and RAB20 knockout mouse peritoneal macrophages stimulated with SiO_2_ after LPS priming. **(H)** IL-1β secretion in THP1 cells with RAB20 siRNA and then stimulated with SiO_2_ after LPS priming. **(I)** Immunoblotting for IL-1β, caspase-1, NLRP3, and β-actin in the supernatants (SN) or cell lysates (cell) of THP1 cells with RAB20 siRNA and then stimulated with SiO_2_ after LPS priming. *p < 0.05; **p < 0.01; ***p < 0.001 (unpaired/two-tailed t test, one-way and two-way ANOVA test). Data are shown as mean ± SEM from three independent experiments.

Next, we investigated the mechanisms by which RAB20 deficiency promotes pulmonary interstitial fibrosis and respiratory dysfunction in our model of silicosis. Since proinflammatory cytokines drive the progression of silicosis, we performed ELISA to measure the cytokine levels in the BALF and found the increased release of IL-1β and IL-18 in mice receiving intratracheal silica crystal suspension (**Figures 3A, B**). Notably, RAB20 deficiency was associated with significantly higher concentrations of IL-1β and slightly higher concentrations of IL-18 in the BALF when compared to WT mice established with silicosis (**Figures 3A, B**). Previous findings had reported that silica crystals induce the release of IL-1β and IL-18 and pulmonary interstitial fibrosis through the NLRP3 inflammasomes (14) (tetracycline ameliorates silica-induced pulmonary inflammation and fibrosis *via* inhibition of caspase-1). We also detected the cleavage of caspase-1, which represents the activation of NLRP3 by immunoblotting in mice lung tissues. In line with previous results, silica crystal promotes caspase-1 cleavage in mice lung tissues (**Figure 3C**). Moreover, deletion of RAB20 markedly increased caspase-1 cleavage in mice lung tissues (**Figure 3C**). Next, *in vitro* macrophage demonstrated that RAB20 deficiency strikingly increased SiO_2_ and MSU-mediated IL-1β release and NLRP3 activation with slightly increased release of IL-18, but failed to affect ATP and nigericin-mediated NLRP3 activation, compared to the stimulated WT macrophage (**Figures 3D–G**). Based on these results, we found that silencing RAB20 in human THP1 cells also increased SiO_2_-mediated IL-1β release and NLRP3 activation (**Figures 3H, I**). These observations clearly suggest that RAB20 deficiency promotes the activation of NLRP3 inflammasome upon silica crystal stimulation.

### Knockout of NLRP3 blocks RAB20 deficiency-induced proinflammatory cytokine release

**Figure 4:**
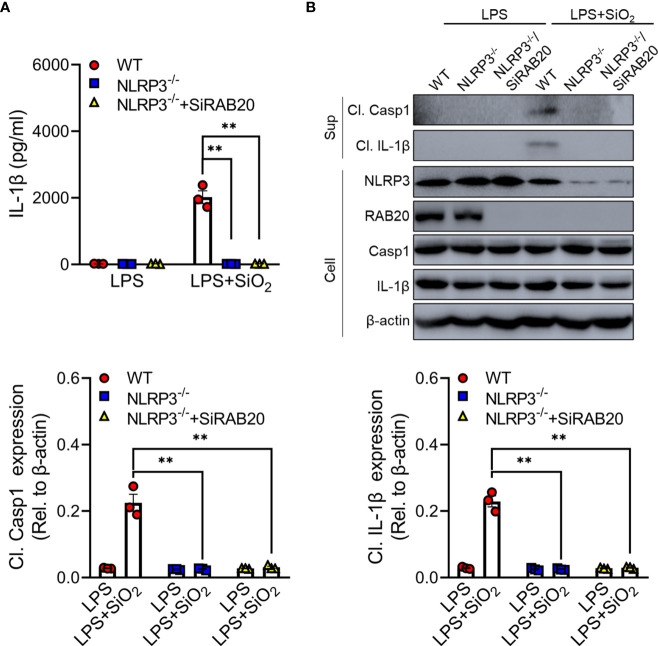
Knockout of NLRP3 blocks RAB20 deficiency-induced proinflammatory cytokine release **(A)** IL-1β secretion in wild type, NLRP3 knockout, and NLRP3 knockout/RAB20 knockdown peritoneal macrophages stimulated with SiO_2_ after LPS priming. **(B)** Immunoblotting for IL-1β, caspase-1, NLRP3, and β-actin in the supernatants (SN) or cell lysates (cell) of wild type, NLRP3 knockout, and NLRP3 knockout/RAB20 knockdown mouse peritoneal macrophages stimulated with SiO_2_ after LPS priming. **p < 0.01 (unpaired/two-tailed t test, one-way and two-way ANOVA test). Data are shown as mean ± SEM from three independent experiments.

To further clarify whether RAB20 deficiency mediated a proinflammatory role *via* NLRP3 inflammasome, RAB20 siRNAs were transfected into NLRP3 knockout peritoneal macrophage to co-inhibit the expression of RAB20 and NLRP3. Compared to WT macrophages, NLRP3 knockout significantly prevented silica crystal-induced IL-1β release and caspase-1 cleavage (**Figures 4A, B**). Moreover, NLRP3 knockout also inhibits RAB20 silencing-mediated IL-1β release and caspase-1 cleavage (**Figures 4A, B**). These observations clearly suggest that knockout of NLRP3 blocks RAB20 deficiency-induced proinflammatory cytokine release.

### RAB20 knockout induced silica crystal-induced NLRP3 inflammasome activation by triggering lysosomal rupture

**Figure 5:**
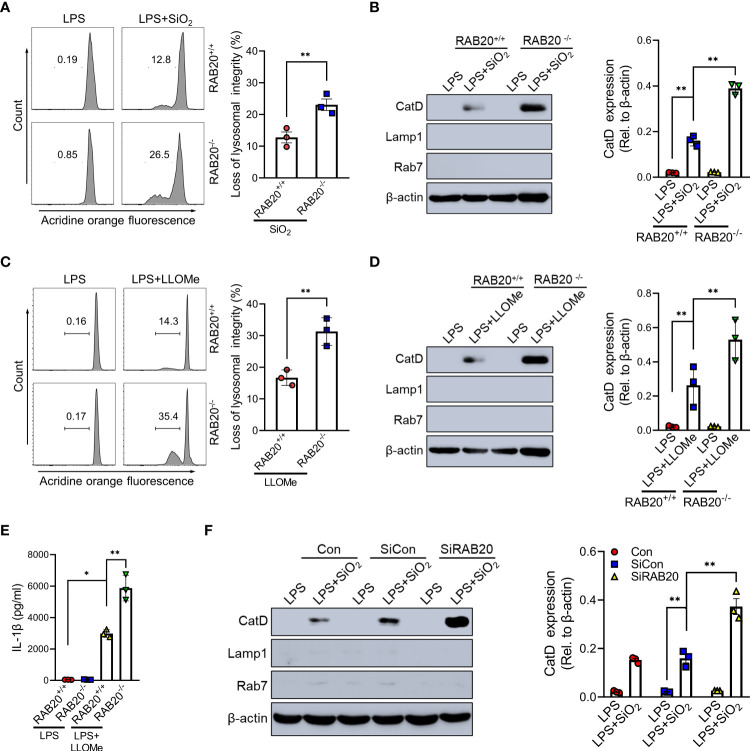
RAB20 knockout induced silica crystal-induced NLRP3 inflammasome activation by triggering lysosomal rupture. **(A)** Immunoblotting for Cathepsin D, RAB7, Lamp1, and β-actin in the cytosolic fraction from RAB20^+/+^ and RAB20^-/-^ mouse peritoneal macrophages stimulated with SiO_2_ after LPS priming. **(B)** Flow cytometry of RAB20^+/+^ and RAB20^-/-^ mouse peritoneal macrophages that stained with acridine orange and then treated with SiO_2_ after LPS priming. **(C)** Immunoblotting for Cathepsin D, RAB7, Lamp1, and β-actin in the cytosolic fraction from RAB20^+/+^ and RAB20^-/-^ mouse peritoneal macrophages stimulated with Leu-Leu-OMe after LPS priming. **(D)** Flow cytometry of RAB20^+/+^ and RAB20^-/-^ mouse peritoneal macrophages that stained with acridine orange and then treated with Leu-Leu-OMe after LPS priming. **(E)** IL-1β secretion in RAB20^+/+^ and RAB20^-/-^ mouse peritoneal macrophages stimulated with Leu-Leu-OMe after LPS priming. **(F)** Immunoblotting for Cathepsin D, RAB7, Lamp1, and β-actin in the cytosolic fraction from THP1 cells with RAB20 siRNA and then stimulated with SiO_2_ after LPS priming. *p < 0.05; **p < 0.01 (unpaired/two-tailed t test, one-way and two-way ANOVA test). Data are shown as mean ± SEM from three independent experiments.

To confirm that priming with RAB20 deficiency promotes silica crystal-induced lysosomal rupture, we isolated the cytoplasmic protein from macrophages and detected the quantity of lysosomal degradation enzyme cathepsin D, a lysosomal protein that is released to the cytoplasm, thus triggering lysosomal rupture. Compared to WT macrophage, silica crystal stimulation induced much more levels of cathepsin D in the cytoplasmic compartment of RAB20 knockout macrophage (**Figure 5A**). In addition, we used acridine orange (AO) to quantitatively measure lysosomal integrity and found that RAB20 deficiency increased silica crystal-induced lysosomal damage in macrophages (**Figure 5B**). To provide further evidence that RAB20 deficiency aggravates silica crystal-induced NLRP3 inflammasome activation and IL-1β release by promoting lysosomal rupture, WT and RAB20-deficient macrophages were incubated with L-leucyl-L-leucine methyl ester (Leu-Leu-OMe), a functionalized dipeptide that converts to a membranolytic compound by the lysosomal enzyme dipeptidyl peptidase I to disrupt lysosome (14, 18). RAB20 deficiency increased Leu-Leu-OMe-induced lysosomal damage and leakage of cathepsin D to the cytosol (**Figures 5C, D**). Accordingly, RAB20 deficiency markedly promotes Leu-Leu-OMe-mediated release of IL-1β in macrophages (**Figure 5E**). In addition, silencing RAB20 in human THP1 cell also induced much more leakage of cathepsin D in the cytoplasmic compartment (**Figure 5F**). Taken together, these findings proved that the RAB20 deficiency enhanced silica crystal-induced NLRP3 inflammasome activation by promoting lysosomal rupture.

### RAB20 knockout reduces the ratio of silica crystal/phagosomal area

**Figure 6:**
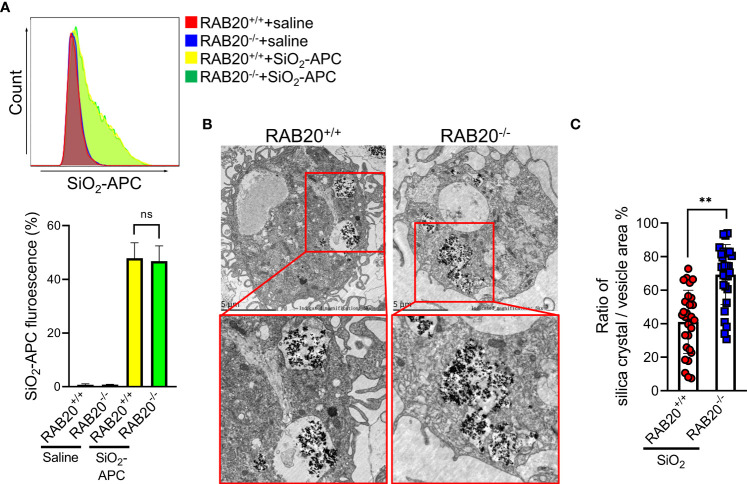
RAB20 knockout reduces the ratio of silica crystal/phagosomal area. **(A)** The Alex Flour 594 fluorescence intensity of wild type and RAB20 knockout mouse peritoneal macrophages stimulated with Alex Flour 594-binding SiO_2_ after LPS priming. **(B)** Representative TEM images of wild type and RAB20 knockout mouse peritoneal macrophages stimulated with SiO_2_ after LPS priming. **(C)** The statistical data of the ratio of silica crystal/phagocytic vesicle area in wild type and RAB20 knockout mouse peritoneal macrophages stimulated with SiO_2_ after LPS priming. **p < 0.01; no statistical difference (unpaired/two-tailed t test, one-way and two-way ANOVA test). Data are shown as mean ± SEM from three independent experiments. Scale bar represents 5 μm.

We next investigated the mechanisms by which RAB20 deficiency promotes silica crystal-induced lysosomal damage. As shown by both flow cytometry and fluorescent microscopy, RAB20 deficiency did not affect the phagocytosis of Alexa Fluor 594-labeled silica crystals in macrophages (**Figure 6A**). This result suggested that RAB20 as a lysosomal protein does not influence the phagocytic function of endosome/lysosome. During *Mycobacterium tuberculosis* infection, RAB20 facilitates the generation of spacious phagosomes and thereby maintains phagosomal integrity in macrophages (19). To further explore how RAB20 deficiency promotes silica crystal-induced lysosomal membrane injury, WT and RAB20-deficient macrophages were stimulated with silica crystals and then subjected to transmission electron microscope (TEM) analysis. We observed that the volume of phagosomal area is slightly increased after silica crystal stimulus to avoid silica crystal-induced mechanical damage. However, RAB20-deficient macrophage inhibits the spacious phagosome formation to increase the ratio calculated as silica crystals/phagosomal area (**Figures 6B, C**). These observations clearly suggest that RAB20 deficiency increased the contact between silica crystals and lysosomal membrane and thereby injures lysosomal membranes from silica crystal-induced mechanical damage.

### Reducing the contact between phagocytic membrane and silica crystals inhibits silica crystal-induced lysosomal rupture and NLRP3 activation during RAB20 deficiency

**Figure 7:**
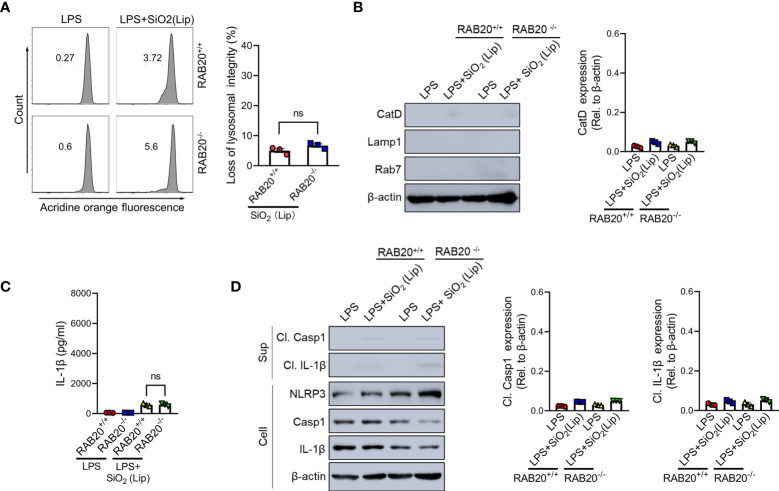
Reducing the contact between phagocytic membrane and silica crystals inhibits silica crystal-induced lysosomal rupture and IL-1β release during RAB20 deficiency. **(A)** Immunoblotting for Cathepsin D, RAB7, Lamp1, and β-actin in the cytosolic fraction from RAB20^+/+^ and RAB20^-/-^ mouse peritoneal macrophages stimulated with liposome-coated silica crystals after LPS priming. **(B)** Flow cytometry of RAB20^+/+^ and RAB20^-/-^ mouse peritoneal macrophages that stained with acridine orange and then treated with liposome-coated silica crystals after LPS priming. **(C)** IL-1β secretion in RAB20^+/+^ and RAB20^-/-^ mouse peritoneal macrophages stimulated with liposome-coated silica crystals after LPS priming. **(D)** Immunoblotting for IL-1β, caspase-1, RAB20, and β-actin in the supernatants (SN) or cell lysates (cell) of RAB20^+/+^ and RAB20^-/-^ mouse peritoneal macrophages that stimulated with liposome-coated silica crystals after LPS priming. NS, no statistical difference (unpaired/two-tailed *t*-test, one-way and two-way ANOVA test). Data are shown as mean ± SEM from three independent experiments.

To provide evidence to support the conclusion that RAB20 deficiency promotes the contact between silica crystals and phagocytic membrane that inhibits silica lysosomal damage and NLRP3 activation, the silica crystals were encapsulated in liposomes to partially and transiently reduce its direct contact with phagosomal membranes. As revealed by acridine orange staining, encapsulation of silica crystals by liposomes markedly reduced the lysosomal damage in RAB20 deficiency and WT macrophages (**Figures 7A, B**). In addition, encapsulation of silica crystals did not further induce cleavage of caspase-1 and release of IL-1β in RAB20 deficiency and WT macrophages (**Figures 7C, D**). Together, these observations clearly suggest that RAB20 deficiency increases the contact between silica crystal and lysosomal membrane to induce mechanical damage and thereby promotes lysosomal damage and NLRP3 activation.

### Cathepsin B involved in RAB20-mediated inflammatory factor release

**Figure 8:**
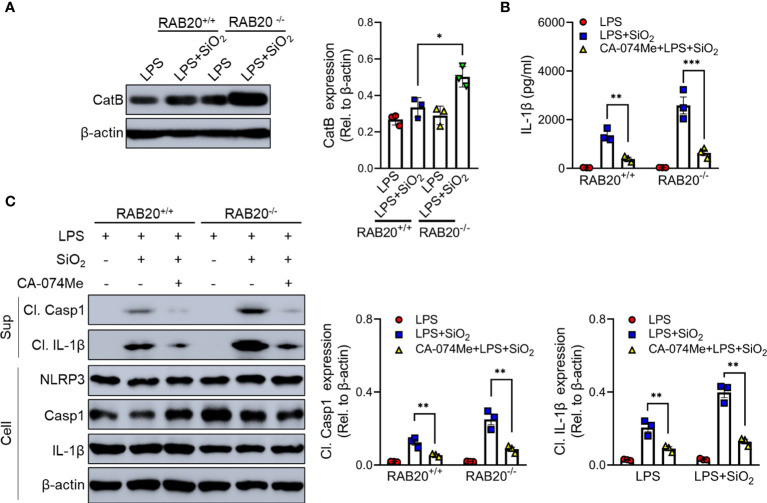
RAB20 knockout promotes the leakage of cathepsin B from lysosome to cytoplasm. **(A)** Immunoblotting for Cathepsin B and β-actin in the cytosolic fraction from RAB20^+/+^ and RAB20^-/-^ mouse peritoneal macrophages stimulated with silica crystals after LPS priming. **(B)** IL-1β secretion in wild type and RAB20 knockout peritoneal macrophages stimulated with SiO_2_ after CA-074Me and LPS priming. **(C)** Immunoblotting for IL-1β, caspase-1, NLRP3, and β-actin in the supernatants (SN) or cell lysates (cell) of wild type and RAB20 knockout mouse peritoneal macrophages stimulated with SiO_2_ after CA-074Me and LPS priming. *p < 0.05; **p < 0.01; ***p < 0.001 (unpaired/two-tailed t test, one-way and two-way ANOVA test). Data are shown as mean ± SEM from three independent experiments.

Cathepsin B as a lysosomal protease is mainly located in the lysosome. During lysosomal damage, cathepsin B leaks from the lysosome to the cytoplasm and induces NLRP3 activation (20). We found that RAB20 deficiency promotes leakage of cathepsin B from the lysosome to the cytoplasm (**Figure 8A**). Moreover, selective cathepsin B inhibitor CA-074Me were used to inhibit the activity of cathepsin B in macrophage (21). Our results showed that CA-074Me partially inhibited SiO_2_-mediated IL-1β release and NLRP3 activation in RAB20 knockout macrophage (**Figures 8B, C**), suggesting that cathepsin B is an important medium that participated in RAB20 deficiency-induced excessive NLRP3 activation.

## Methods

### Clinical human samples

Patients were diagnosed with or without silicosis according to an occupational history of exposure to silica crystal, associated with radiologic studies with characteristic findings (standard chest x-ray with a profusion ≥1/1 according to the ILO classification) and exclusion of other possible entities (22, 23). A total of 42 patients including exposure patients (*n* = 19) and silicosis patients (*n* = 23) from the Hunan Prevention and Treatment Institute for Occupational Diseases from August 2019 to December 2020 met the above conditions (**Supplementary Tables 1, 2**). The miners that were diagnosed with silicosis will undergo bronchoscopy examination, and those who suffered from tuberculosis and lung cancer will be excluded. The bronchoalveolar lavage fluid was obtained immediately after lung washing therapy. Informed consent was conferred by the patients or their families, and this study was approved by the research ethics committee of the Third Xiangya Hospital of Central South University.

### Analysis of bronchoalveolar lavage fluid cells by single-cell RNA-seq

The feature-barcode matrix was downloaded from GEO: GSE174725. Low-quality cells were excluded from the analysis if the mitochondrial genes represented >15% or if the number of features in a cell was <200. Count values were normalized, log-transformed, and scaled using the Seurat R package v3.6.

### Mouse studies

Experimental protocols were approved by the Institutional Animal Care and Use Committees of the Central South University. WT mice were purchased from Jackson Laboratory. RAB20^-/-^ were generated by the CRISPR/Cas9-mediated genome editing (GemPharmatech Co. Ltd). Briefly, the vectors that encode Cas9 and guide RNA were transcribed into mRNA and gRNA *in vitro*, and then injected into the fertilized eggs that were transplanted into pseudo-pregnant mice. The targeted genome of F0 mice was amplified with PCR and sequenced, and the offspring were crossed with wild-type C57BL/6 mice (GemPharmatech Co. Ltd) to obtain the RAB20^+/-^ mice. The F1 RAB20^+/-^ mice were further crossed with wild-type C57BL/6 mice for at least RAB20^+/+^, RAB20^+/-^, or RAB20^-/-^ generations. Mice were genotyped by PCR analysis of tail DNA followed by sequencing and the resulting RAB20^+/-^ mice were crossed to generate RAB20^+/+^ and RAB20^-/-^ mice [RAB20 typing sequence 1 (Common): F: ATCCAACCACAGGTGGAATAGC, R: TGATGCTGGGAATTCACCTG and RAB20 typing sequence 2 (Mutation): F: AACCTTGTTTGACCTGGTGGTAC, R: TAGTGACTGCAGAGTCAAGACCAG].

All animals were maintained in a specific pathogen-free environment at the Department of Laboratory Animals of Central South University. In the current study, we used WT littermates as the controls for the transgenic mice. All experimental animal protocols were approved by the Institutional Animal Care and Use Committees of Central South University.

### Silica crystals and liposome-coated silica crystals

The silica crystals with a diameter of less than 0.1 μm used in cell stimulation was purchased from *In vivo*Gen, and the ultrafine silica powder MIN-U-SIL 5 (a diameter of less than 1 μm) used in mice was purchased from U.S. SILICA. To obtain nano silica crystals with a diameter of less than 10 nm used in our experiment, the silica crystal suspension was subjected to ultrasound at 100 Hz to scatter the silica crystal dusts and a dialysis was performed using regenerated cellulose (RC) membrane (5000D, Coolaber). To obtain liposome-coated silica crystals, the silica crystals were coated with P3000 (Invitrogen) and then encapsulated by liposome Lipofectamine3000 (Invitrogen).

### Silicosis model

Male mice that were 25 to 30 g in weight were given silica crystals (200 mg/kg) *via* single orotracheal atomizing injection. Mice were exposed to silica crystals for 4 weeks. Mice in the control group were exposed to saline. At the end of the time point, mice were anesthetized and micro-CT scanning or lung function test was performed.

### Macrophage preparation and stimulation

Mouse peritoneal macrophages were isolated and cultured as described previously (24). Briefly, mice (8 weeks old) were injected with 3 ml of sterile 3% thioglycollate broth intraperitoneally to elicit the formation of peritoneal macrophages. After 72 h, cells were collected *via* lavage of the peritoneal cavity with 10 ml of RPMI 1640 medium (GIBCO). Then, cells were centrifuged at 800 rpm and were resuspended in RPMI 1640 medium supplemented with 10% fetal bovine serum (GIBCO) and 1% penicillin–streptomycin (Thermo Fisher). Peritoneal macrophages (10^6^ cells per well) were plated in 12-well plates and then primed with ultra-pure LPS (100 ng/ml) for 4 h, and then stimulated with SiO_2_ crystal (20 μg/ml), APC-connected silica crystals (20 μg/ml), or liposome-coated silica crystals (20 μg/ml) for 6 h. To induce lysosomal disruption, peritoneal macrophages were primed with ultra-pure LPS (1 ng/ml) for 4 h and then were stimulated with Leu-Leu-OMe·HCl (1,000 μM, Chem-Impex International) for 5 h. To inhibit the activity of cathepsin B, peritoneal macrophages were primed with 100 μM CA-074Me (Selleck) overnight before ultra-pure LPS priming.

### Lung function

Before the test, mice were anesthetized with sodium pentobarbital (50 mg/kg). After attaining complete relaxation, all animals were operated on using a standard catheter (CNS5002) provided by Buxco equipment. After finishing the tracheostomy and intubation, mice were placed in a body plethysmograph connected to a computer-controlled ventilator to detect the lung function. The Buxco pulmonary function testing system (Buxco, Sharon, Connecticut, CT, USA) was used to analyze dynamic compliance (Cdyn), tidal volume (TV), minute volume (MV), and respiratory resistance index (RI) of the mice.

### H&E and Masson staining

The lung tissues were sequentially fixed in 4% neutral formalin solution for 48 h, dehydrated in 30% sucrose, embedded in paraffin, and cut into 4-μm sections. Hematoxylin and eosin staining and Masson staining were performed to observe the pathologic and morphologic characteristics of the tissues. The area fraction of granuloma was observed by the random non-coincident microscopic fields at a magnification of ×200. The collagen fiber thresholds were established after the fibers were easily identified as birefringent bands by increasing the contrast value. The collagen fiber area can be calculated by digital densitometric recognition. During the measurements of collagen fibers, the bronchi and blood vessels should be carefully avoided. The area occupied by fibers was divided by tissue area and expressed as the fraction area of collagen fibers.

### RNAi silencing

For siRNA silencing of RAB20, mouse peritoneal macrophages were cultured in 12-well plates at 5 × 10^5^ cells/well at the time of transfection. siRNA transfection was performed using the Lipofectamine RNAi MAX Transfection Reagent by following the manufacturer’s instructions. Seventy-two hours after transfection, cells were primed with ultra-pure LPS and then stimulated with silica crystal. The mouse siRNA target sequences are GCCGCTATCATCCTTACAT (RAB20) and TTCTCCGAACGTGTCACGT (negative control). The human siRNA target sequences are CCTCTTTGAAACCTTGTTT (RAB20) and CGTACGCGGAATACTTCGA (negative control). The silencing efficiency was examined by Western blot using the corresponding antibodies.

### Elisa

The concentration of IL-1β and IL-18 in bronchoalveolar lavage fluid and cell culture supernatant samples were analyzed using IL-1β ELISA kits and IL-18 ELISA kits (Invitrogen).

### Immunoblotting

The supernatant proteins from cell-free supernatants were extracted by methanol–chloroform precipitation. The lung tissue and cell protein were obtained by SDS extracts. The protein samples were separated through SDS-PAGE and then transferred onto PVDF membranes (Millipore). Mouse caspase-1 antibody (Abcam) was used at 1:1,000 dilution. Mouse IL-1β antibody (R&D Systems) was used at 1:1,000 dilution. Mouse NLRP3 antibody (adipogen) was used at 1:1,000. Mouse LAMP1 antibody (Proteintech) was used at 1:1,000 dilution. Mouse RAB7 (CST) was used at 1:1,000 dilution. Mouse cathepsin D antibody (Abcam) was used at 1:1,000 dilution. Blots were normalized to β-actin expression.

### Flow cytometry

For evaluation of lysosomal damage, macrophage cells were pre-incubated with 1 μg/ml acridine orange for 15 min to stain the endosome/lysosome, then washed three times with PBS and subsequently stimulated as indicated using the stimulus. Lysosomal rupture can be assessed and quantized by loss of emission fluorescence at 600–650 nm using flow cytometry. All experiments of flow cytometry were performed on an LSRII cytometer (BD Biosciences). Data were acquired by DIVA (BD Biosciences) and analyzed by FlowJo software (Tree Star Inc., Ashland, OR).

### Quantification and statistical analysis

All data were analyzed using GraphPad Prism software (version 5.01). Data were analyzed using Student’s *t*-test and were used for comparison between two groups or one-way ANOVA followed by *post-hoc* Bonferroni test for multiple comparisons. *p*-value < 0.05 was considered statistically significant for all experiments. All values are presented as the mean ± SD.

## Discussion

This work establishes the critical role of RAB20 in preventing silica crystal-induced inflammatory responses and pulmonary interstitial fibrosis, a process that resembles the immunopathology of clinical silicosis. Knockout of RAB20 induced severe silicosis development by significantly promoting NLRP3 inflammasome activation and IL-1β release. These findings are based on the following results: (i) RAB20 gene expression was decreased in silicosis patients; (ii) genetic ablation of RAB20 aggravates the development of silicosis by increasing NLRP3 inflammasome activation and IL-1β release; (iii) RAB20 promoted silica crystal-induced NLRP3 inflammasome activation and IL-1β release through inducing lysosomal damage; (iv) RAB20 knockout reduces the ratio of silica crystal/phagosomal area to promote lysosomal damage; and (v) reducing the contact between phagocytic membrane and silica crystals inhibits silica crystal-induced lysosomal rupture and NLRP3 activation during RAB20 deficiency.

Accumulating evidence shows that deregulated pulmonary NLRP3 immune signaling drives the development and progression of silicosis (14–16). Alveolar macrophages are the major reason to induce lung inflammation in response to both infectious and non-infectious agents. Phagocytosis of the inhaled silica crystals by alveolar macrophages results in lysosomal damage, which elicits the activation of NLRP3 inflammasome to mediate the activation of caspase-1 and the subsequent maturation of proinflammatory cytokines including interleukin (IL)-1β in murine silicosis models. Deletion of the genes encoding NLRP3 inflammasome components markedly attenuates silica crystal-induced granulomatous lung inflammation and pulmonary fibrosis (15, 16). In this study, we found that RAB20 deficiency can activate NLRP3 inflammasome by promoting lysosomal disruption (25), simultaneous knockdown/knockout of RAB20 and NLRP3 in macrophage can effectively reverse SiO_2_-induced inflammatory factor release, but RAB20/NLRP3 double knockout mice must still be created to ensure the role of the RAB20/NLRP3 pathway in silicosis. Studies have confirmed that silica crystal, asbestos, and aluminum crystal are all NLRP3 activators by mediating the lysosomal damage (15). Cathepsin B and calcium ion released from lysosomal disruption promote the NLRP3 inflammasome by promoting ASC oligomerization (26). In spite of this, the molecular mechanism of NLRP3 activation in silicosis that causes lysosome damage remains unknown.

Rab family small GTPases that belong to the Ras superfamily are the key target in membrane traffic, especially in the lysosome, in all eukaryotic cells (27). Like other small GTPases, Rab cycles between two guanine nucleotide binding states, a GTP-bound active state and a GDP-bound inactive state. In its active state, each Rab interacts with a specific effector molecule and controls a specific membrane traffic pathway. Among RAB20, a submembrane of the Rab family is mainly located in the lysosomal membrane and regulates intracellular vesicular trafficking and endosomal membrane influx (19). Previous studies found out that the lack of RAB20 can be beneficial for *Mycobacterium tuberculosis* (Mtb) replication in macrophage (19). Mtb mainly infects the pulmonary respiratory tract and causes tuberculosis, which is the most important complication of silicosis patients. However, whether RAB20 deficiency also influences the process of endosome-mediated phagocytosis of silica crystals remains unclear.

Though RAB20 deficiency does not affect the phagocytosis of silica crystals by macrophages, RAB20 deficiency inhibits the self-protection of lysosome by repressing spacious phagosome formation with silica crystals’ stimulation and thereby increases the ratio of silica crystals/phagosomal area. Presumably, this process significantly increases the frequency of contact between silica crystals and phagosomal membranes, and thereby injures the integrity of silica crystal-containing phagosomes. This process promotes the leakage of cathepsin B, an important activator of NLRP3 inflammasome, from lysosome to cytosol. In addition, combined with our finding that impaired RAB20 expression promotes the development of silicosis and previous reports that loss of RAB20 gene promotes the replication of Mtb, these results may provide an explanation for the previously unsolved mystery why tuberculosis risk is associated with severity of silicosis.

However, due to the potential gene expression and cell function difference between peritoneal macrophages and bronchoalveolar alveolar lavage fluid macrophages, we still need to further prove this conclusion by using bronchoalveolar alveolar lavage-derived macrophages.

Taken together, this study provides a novel link between RAB20 deficiency and susceptibility of silicosis with implications for the prevention of this occupational disease.

## Data availability statement

The datasets presented in this study can be found in online repositories. The name of the repository and accession number can be found below: NCBI Gene Expression Omnibus, GSE174725.

## Ethics statement

The studies involving human participants were reviewed and approved by Research ethics committee of the Third Xiangya Hospital of Central South University. The patients/participants provided their written informed consent to participate in this study. The animal study was reviewed and approved by Institutional Animal Care and Use Committees of the Central South University.

## Author contributions

FL conceived the project and designed experiments and wrote the paper. ZP supervised the study, designed experiments, performed the experiments, analyzed the data, and wrote the paper. MD, KZ, and YT performed the experiments and assisted in data interpretation. All authors contributed to the article and approved the submitted version.

## Acknowledgments

We thank Qianqian Xue and Ling Li for managing mouse colonies and research assistance. This work was supported by the National Natural Science Foundation of China (No. 81971893 to YT and No. 81700127 to FL), the Natural Science Foundation of Hunan Province (No. 2020JJ5860 to FL and No. 2020JJ5864 to ZP), the Fundamental Research Funds for the Central Universities of Central South University 2021zzts0402, and the Hunan Provincial Innovation Foundation For Postgraduate CX20210374.

## Conflict of interest

The authors declare that the research was conducted in the absence of any commercial or financial relationships that could be construed as a potential conflict of interest.

## Publisher’s note

All claims expressed in this article are solely those of the authors and do not necessarily represent those of their affiliated organizations, or those of the publisher, the editors and the reviewers. Any product that may be evaluated in this article, or claim that may be made by its manufacturer, is not guaranteed or endorsed by the publisher.
