# Supplementary material for: RAB20 deficiency promotes the development of silicosis via NLRP3 inflammasome
Source: Front Immunol. 2022 Sep 5;13:967299. doi: 10.3389/fimmu.2022.967299 (PMC9484360; doi:10.3389/fimmu.2022.967299)
Supplement: Supplementary file 1 [file Table_1.docx]

**Supplemental table1**

| **ID** | **Age**  **(Years)** | **Gender** | **Clinical**  **grading** | **Smoking**  **/Vape** | **Working**  **year** |
| --- | --- | --- | --- | --- | --- |
| Exposure1 | 44 | Male | Con | Yes | 22 |
| Exposure2 | 55 | Male | Con | Yes | 26 |
| Exposure3 | 47 | Male | Con | Yes | 14 |
| Exposure4 | 39 | Male | Con | NO | 17 |
| Exposure5 | 47 | Male | Con | Yes | 16 |
| Exposure6 | 56 | Male | Con | Yes | 31 |
| Exposure7 | 50 | Male | Con | Yes | 29 |
| Exposure8 | 53 | Male | Con | Yes | 19 |
| Exposure9 | 55 | Male | Con | Yes | 31 |
| Exposure10 | 48 | Male | Con | Yes | 11 |
| Exposure11 | 44 | Male | Con | NO | 9 |
| Exposure12 | 54 | Male | Con | Yes | 26 |
| Exposure13 | 46 | Male | Con | Yes | 17 |
| Exposure14 | 39 | Male | Con | Yes | 13 |
| Exposure15 | 54 | Male | Con | Yes | 26 |
| Exposure16 | 52 | Male | Con | Yes | 27 |
| Exposure17 | 45 | Male | Con | Yes | 23 |
| Exposure18 | 52 | Male | Con | NO | 32 |
| Exposure19 | 42 | Male | Con | Yes | 17 |
| Exposure20 | 46 | Male | III | Yes | 25 |
| Exposure21 | 51 | Male | III | Yes | 26 |
| Exposure22 | 47 | Male | III | Yes | 18 |
| Exposure23 | 46 | Male | III | NO | 14 |
| Silicosis1 | 58 | Male | III | Yes | 34 |
| Silicosis2 | 44 | Male | III | NO | 13 |
| Silicosis3 | 48 | Male | III | Yes | 22 |
| Silicosis4 | 55 | Male | II | Yes | 10 |
| Silicosis5 | 50 | Male | II | Yes | 19 |
| Silicosis6 | 48 | Male | II | Yes | 22 |
| Silicosis7 | 40 | Male | II | Yes | 20 |
| Silicosis8 | 40 | Male | II | NO | 15 |
| Silicosis9 | 49 | Male | II | Yes | 14 |
| Silicosis10 | 53 | Male | I | Yes | 28 |
| Silicosis11 | 52 | Male | I | Yes | 30 |
| Silicosis12 | 55 | Male | I | Yes | 21 |
| Silicosis13 | 48 | Male | I | NO | 26 |
| Silicosis14 | 41 | Male | I | Yes | 15 |
| Silicosis15 | 44 | Male | I | Yes | 24 |
| Silicosis16 | 52 | Male | I | NO | 18 |
| Silicosis17 | 55 | Male | I | Yes | 20 |
| Silicosis18 | 48 | Male | I | NO | 28 |
| Silicosis19 | 41 | Male | I | Yes | 11 |

**Table S1. The clinical information of exposure miners and silicosis patients.**
