# Supplementary material for: RAB20 deficiency promotes the development of silicosis via NLRP3 inflammasome
Source: Front Immunol. 2022 Sep 5;13:967299. doi: 10.3389/fimmu.2022.967299 (PMC9484360; doi:10.3389/fimmu.2022.967299)
Supplement: Supplementary file 2 [file Table_2.docx]

**Supplemental table2**

| **Groups** | **Age (Years)**  **Average** | **Working year**  **Average** |
| --- | --- | --- |
| Exposure | 48.5 (±7.5) | 21.4 (±12.4) |
| Silicosis | 48.3 (±7.3) | 20.6 (±9.6) |

**Table S2. The average age and working year of exposure miners and silicosis patients.**
